# Supplementary material for: A framework for subsurface monitoring by integrating reservoir simulation with time-lapse seismic surveys
Source: Sci Rep. 2023 Aug 22;13:13661. doi: 10.1038/s41598-023-40548-0 (PMC10444811; doi:10.1038/s41598-023-40548-0)
Supplement: Supplementary file 1 — Supplementary Information. [file 41598_2023_40548_MOESM1_ESM.pdf]

A framework for subsurface monitoring by integrating reservoir simulation with time-lapse seismic surveys: Supplementary Information.

Johno van IJsseldijk[1,*], Hadi Hajibeygi[1], and Kees Wapenaar[1]

[1]Delft University of Technology, Department of Geoscience and Engineering, Delft, The Netherlands
[*]J.E.vanIJsseldijk@tudelft.nl

Derivation of the Marchenko-based isolation of the reservoir response.

The Marchenko method relies on two Green's function representations that relate the Green's functions to the focusing functions via reflection response R. The extrapolated Green's function representations read as follows [1]:

$$U^{-,+}(\mathbf{x}_R,\mathbf{x}'_S,t)+v^-(\mathbf{x}_R,\mathbf{x}'_S,t)=\int_{\mathbb{S}_0}R(\mathbf{x}_R,\mathbf{x}_S,t)*v^+(\mathbf{x}_S,\mathbf{x}'_S,t)d\mathbf{x}_S, \tag{1}$$

and

$$U^{-,-}(\mathbf{x}_R,\mathbf{x}'_S,-t)+v^+(\mathbf{x}_R,\mathbf{x}'_S,t)=\int_{\mathbb{S}_0}R(\mathbf{x}_R,\mathbf{x}_S,-t)*v^-(\mathbf{x}_S,\mathbf{x}'_S,t)d\mathbf{x}_S. \tag{2}$$

Here, $*$ denotes a convolution and the right-hand side integrates over the source positions $\mathbf{x}_S$ at the acquisition surface $\mathbb{S}_0$. Moreover, $\mathbf{x}_R$ and t denote the receiver coordinate at the acquisition surface and time, respectively. $U^{-,\pm}(\mathbf{x}_R,\mathbf{x}'_S,t)$ is the Green's function with an up-going receiver field from an up $(-)$ or down-going $(+)$ source field at a focal level in the subsurface, extrapolated to $\mathbf{x}'_S$ at the surface. The extrapolated up- and down-focusing functions (i.e. $v^-(\mathbf{x}_R,\mathbf{x}'_S,t)$ and $v^+(\mathbf{x}_R,\mathbf{x}'_S,t)$) are defined in a truncated medium, which is the same as the actual medium above an arbitrary focal level and homogeneous below that level. In the actual medium these focusing functions let the wavefield converge to the focal point, creating a virtual source that produces the Green's functions between the focal depth and the surface.
Assuming the reflection response R is known (from seismic reflection measurements at the surface), equations 1 and 2 have four unknowns, but can be solved by adding a causality constraint, which takes advantage of the fact that the focusing functions and Green's functions are separable in time [2]. In order to achieve this separation an estimate of the two-way traveltime (twt) from $\mathbb{S}_0$ to the focal depth and back is required. This estimate can for example be obtained from a smooth velocity model. By limiting equations 1 and 2 between $t=0s$ and this twt, the Green's functions in the left-hand side vanish [1]; the resulting equations are known as the extrapolated Marchenko equations:

$$v^-(\mathbf{x}_R,\mathbf{x}'_S,t)=\Theta\int_{\mathbb{S}_0}R(\mathbf{x}_R,\mathbf{x}_S,t)*v^+(\mathbf{x}_S,\mathbf{x}'_S,t)d\mathbf{x}_S, \tag{3}$$

and

$$v^+(\mathbf{x}_R,\mathbf{x}'_S,t)-v^+_d(\mathbf{x}_R,\mathbf{x}'_S,t)=\Theta\int_{\mathbb{S}_0}R(\mathbf{x}_R,\mathbf{x}_S,-t)*v^-(\mathbf{x}_S,\mathbf{x}'_S,t)d\mathbf{x}_S. \tag{4}$$

In Equation 4 time-window Θ mutes the Green's functions, while keeping the focusing functions intact. Furthermore, v^+_d represents the direct arrival of the focusing function, which is equal to a delta pulse as follows [3]:

$$v^+_d(\mathbf{x}_R,\mathbf{x}'_S,t)=\delta(\mathbf{x}_{H,R}-\mathbf{x}'_{H,S})\delta(t). \tag{5}$$

where $\mathbf{x}_{H,R}$ and $\mathbf{x}'_{H,S}$ are the horizontal coordinates of $\mathbf{x}_R$ and $\mathbf{x}'_S$, respectively. Subsequently, the reservoir response is extracted from the full seismic response using two-fold application of these equations [4]. To achieve this the subsurface is divided in three units; overburden a, target zone b and underburden c. Where target zone b contains the reservoir response. First, the overburden is removed by finding the Green's functions at a focal level between overburden a and target zone b from equations 1 and 2:

$$U^{-,+}_{a|bc}(\mathbf{x}_R,\mathbf{x}'_S,t)=\Psi\int_{\mathbb{S}_0}R_{abc}(\mathbf{x}_R,\mathbf{x}_S,t)*v^+_{a|bc}(\mathbf{x}_S,\mathbf{x}'_S,t)d\mathbf{x}_S, \tag{6}$$

and

$$U^{-,-}_{a|bc}(\mathbf{x}_R,\mathbf{x}'_S,-t) = \Psi\int_{\mathbb{S}_0} R_{abc}(\mathbf{x}_R,\mathbf{x}_S,-t) * v^{-}_{a|bc}(\mathbf{x}_S,\mathbf{x}'_S,t)d\mathbf{x}_S. \tag{7}$$

Here, Ψ is a time-window complimentary to Θ, which removes the focusing functions from the right hand-side of the equation. The subscript $a|bc$ denotes that the extrapolated Green's functions are retrieved from the full reflection response (R_{abc}) with a focal depth between units a and b. Using these Green's functions, a reflection response free of overburden interactions (R_{bc}) can be acquired by solving [3]:

$$U^{-,+}_{a|bc}(\mathbf{x}_R,\mathbf{x}'_S,t) = -\int_{\mathbb{S}_0} U^{-,-}_{a|bc}(\mathbf{x}_R,\mathbf{x}'_R,t) * R_{bc}(\mathbf{x}'_R,\mathbf{x}'_S,t)d\mathbf{x}'_R. \tag{8}$$

R_{bc} can be obtained from Equation 8 by means of a multi-dimensional deconvolution [MDD, 5]. To completely isolate the reservoir response, equations 3 and 4 are used together with Equation 5 to find focusing functions between the target zone and underburden from this new reflection response:

$$v^{-}_{b|c}(\mathbf{x}_R,\mathbf{x}'_S,t) = \Theta\int_{\mathbb{S}_0} R_{bc}(\mathbf{x}_R,\mathbf{x}_S,t) * v^{+}_{b|c}(\mathbf{x}_S,\mathbf{x}'_S,t)d\mathbf{x}_S, \tag{9}$$

and

$$v^{+}_{b|c}(\mathbf{x}_R,\mathbf{x}'_S,t) - \delta(\mathbf{x}_{H,R}-\mathbf{x}'_{H,S})\delta(t) = \Theta\int_{\mathbb{S}_0} R_{bc}(\mathbf{x}_R,\mathbf{x}_S,-t) * v^{-}_{b|c}(\mathbf{x}_S,\mathbf{x}'_S,t)d\mathbf{x}_S. \tag{10}$$

The subscript $b|c$ denotes that the extrapolated focusing functions are retrieved from the reflection response without overburden (R_{bc}) using a focal depth between units b and c. These focusing functions can then be used to retrieve the reflection response that only contains target zone events (R_b) [6]:

$$v^{-}_{b|c}(\mathbf{x}_R,\mathbf{x}'_S,t) = \int_{\mathbb{S}_0} v^{+}_{b|c}(\mathbf{x}_R,\mathbf{x}'_R,t) * R_b(\mathbf{x}'_R,\mathbf{x}'_S,t)d\mathbf{x}'_R. \tag{11}$$

Again, the isolated reflection response R_b can be retrieved from this equation by MDD. Equation 11 directly follows from the definition of a focusing function in the truncated medium [3].

References

1. van der Neut, J. & Wapenaar, K. Adaptive overburden elimination with the multidimensional Marchenko equation. *Geophysics* **81**, no. 5, T265–T284, DOI: 10.1190/geo2016-0024.1 (2016).
2. Wapenaar, K. *et al.* Marchenko imaging. *Geophysics* **79**, no. 3, WA39–WA57 (2014).
3. Wapenaar, K. *et al.* Marchenko redatuming, imaging, and multiple elimination and their mutual relations. *Geophysics* **86**, no. 5, WC117–WC140, DOI: 10.1190/geo2020-0854.1 (2021).
4. van IJsseldijk, J., van der Neut, J., Thorbecke, J. & Wapenaar, K. Extracting small time-lapse traveltime changes in a reservoir using primaries and internal multiples after Marchenko-based target zone isolation. *Geophysics* **88**, no 2., R135–R143, DOI: 10.1190/geo2022-0227.1 (2023).
5. Broggini, F., Wapenaar, K., van der Neut, J. & Snieder, R. Data-driven Green's function retrieval and application to imaging with multidimensional deconvolution. *J. Geophys. Res. Solid Earth* **119**, 425–441, DOI: https://doi.org/10.1002/2013JB010544 (2014).
6. Wapenaar, K. & Staring, M. Marchenko-based target replacement, accounting for all orders of multiple reflections. *J. Geophys. Res. Solid Earth* **123**, 4942–4964, DOI: 10.1029/2017JB015208 (2018).
